# The predictive role of NLR and PLR for solid non-AIDS defining cancer incidence in HIV-infected subjects: a MASTER cohort study

**DOI:** 10.1186/s13027-015-0032-y

**Published:** 2015-10-05

**Authors:** Elena Raffetti, Francesco Donato, Francesco Castelli, Franco Maggiolo, Giampiero Carosi, Eugenia Quiros-Roldan

**Affiliations:** Department of Medical and Surgical Specialties, Radiological Sciences and Public Health, Unit of Hygiene, Epidemiology and Public Health, University of Brescia, Viale Europa 11, 25123 Brescia, Italy; University Division of Infectious and Tropical Diseases, University of Brescia, Brescia, Italy; Clinical Infectious Diseases, Ospedale Papa Giovanni XXIII of Bergamo, Bergamo, Italy; Fondazione Malattie Infettive e Salute Internazionale, Brescia, Italy

**Keywords:** Cancer, HIV, Inflammation-based biomarkers, NLR, PLR

## Abstract

**Background:**

The neutrophil to lymphocyte ratio (NLR) and platelet to lymphocyte ratio (PLR), two low cost, routinely available inflammatory indices, have been found to be associated with risk of death in patients with solid cancer, in both general population and HIV-positive subjects. However, no study investigated the role of NLR and PLR as predictive of cancer incidence so far.

**Methods:**

The aim of our study was to assess the association of PLR and NLR with risk of developing solid non-AIDS defining cancer (NADC) in HIV-infected subjects. We conducted a multicenter Italian cohort study from 2000 to 2012 including HIV-infected subjects naïve at antiretroviral treatment at enrollment. The associations of NLR and PLR with NADC incidence were evaluated by univariate and multivariate analyses using both time independent and time dependent Cox proportional hazard models.

**Results:**

Thirteen thousand five hundred fifty-nine patients (73.3 % males) with a mean age of 36.0 years (SD 10.0) were included. The median (inter-quartile range) of NLR and PLR at baseline were 1.47 (1.03–2.17) and 109.9 (79.6–155.3), respectively. During a median follow-up of 3.9 years, 337 subjects had a first diagnosis of solid NADC. The crude and age- and gender-standardized incidence rates were 3.57 and 3.91 per 1000 person-years, respectively. No statistically significant association was found between NLR and PLR and NADC incidence, using multivariate models, including also time-dependent Cox models with a cubic-spline for NLR and PLR.

**Conclusion:**

This study does not sustain the hypothesis that NRL and PLR may be useful for predicting the risk of cancer in HIV positive subjects.

**Electronic supplementary material:**

The online version of this article (doi:10.1186/s13027-015-0032-y) contains supplementary material, which is available to authorized users.

## Introduction

Chronic inflammation is thought to be a key mediator of cancer through the mobilization of transcription factors and inflammatory mediators, as recruitment of inflammatory cells, including neutrophils and megakaryocytes, causes neutrophilia and thrombocytosis on the cancer site, leading to tumor promotion, invasion, and metastasis [1]. Accordingly, the serum level of C-reactive protein, a well-known inflammation marker, was associated with increased risk of some cancers according to recent meta-analyses [2–4].

In HIV infected subjects, the incidence of non-AIDS related chronic disorders, particularly cancer, has increased in last decade, even in those with long-term antiretroviral therapy (ART), and it is now higher than the general population [5, 6], possibly due to residual chronic immune activation and inflammation [7–10]. Indeed, higher levels of several inflammatory biomarkers have been found to be associated with increased risk of AIDS and non-AIDS events, including cancer in these patients [11, 12].

The identification of predictors of cancer incidence in HIV-positive subjects in usual care would be useful to identify interventions to decrease the occurrence of these events. The neutrophil to lymphocyte ratio (NLR) and platelet to lymphocyte ratio (PLR) are low cost, routine available, inflammatory indices, which have been demonstrated useful for predicting risk of death in patients with solid cancer in the general population, independently of tumor characteristics [13–15]. In a previous study among HIV-infected subjects, we found that NLR and PLR serum values were predictive of risk of death in subjects with solid non-AIDS defining cancer (NADC), though not in those with solid AIDS defining cancer (ADC) [16]. However, no study evaluated the possible use of NLR and PLR for predicting cancer incidence in HIV-positive subjects, or in the general population, so far, to our knowledge.

The aim of our study was to assess the association of PLR and NLR with risk of solid NADC in a multicenter cohort of HIV-infected subjects.

## Methods

We conducted a retrospective cohort study of HIV-infected patients of the MASTER cohort in follow-up from January 2000 to December 2012, either ART naive or experienced. Inclusion criteria were: age of 18 years and over, and no solid NADC diagnosis before baseline. The baseline was 1st January 2001 for subjects enrolled in MASTER cohort before that date, and the date of enrollment in the cohort for patients who entered in the cohort after that date. The characteristics of the MASTER cohort and the procedures of cancer data collection have been described elsewhere [17]. The following data were retrieved from the electronic database: gender, age, country of origin, HIV exposure group, date of enrolment in the cohort, viral hepatitis C or B co-infection, ART, AIDS event and cancer occurrence. Moreover, the following parameters, measured within 6 months from the diagnosis of cancer, were retrieved: HIV-RNA, CD4 cell count, CD8 cell count, neutrophil, lymphocyte and platelet count.

The inflammatory factors evaluated were NLR and PLR, considered as continuous and dichotomized according to their median.

The primary outcome was the incidence of NADCs, which were coded according to the international classification of diseases (ICD), 9th and 10th revisions [18]. Hematological cancers (ICD-10 code, from 81 to 96) and solid ADCs (Kaposi sarcoma and invasive cervical carcinoma, ICD-10 code 46 and 53, respectively) were excluded from the analysis.

The study was conducted in accordance with the guidelines of the Declaration of Helsinki and the principles of Good Clinical Practice. The study protocol was approved by the local ethics committees. Informed consent was obtained by all patients enrolled.

### Statistical analysis

Observation time was calculated from study inclusion until cancer occurence, death, last follow-up visit or 31st December 2012.

The differences in demographic, clinical and pathological features between losses to follow-up and non-losses to follow-up were tested using common statistical methods for median and proportion comparisons.

The age- and gender-adjusted NADC incidence rates were calculated dividing the number of observed cases by the corresponding person-years at risk, using the direct method of standardization, truncated at 65 years-old, with the European population as the standard, according to calendar period [19]. All the rates were expressed per 1000 person-years.

The associations of NLR, PLR with cancer incidence were evaluated by univariate and multivariate analysis using both time independent and time dependent Cox proportional hazard models, which provided estimates of hazard ratios (HRs), their 95 % confidence intervals (95 % CIs) and p-values. In time dependent regression models, the study period was divided into intervals of 1-year duration. Gender, age at enrolment, intravenous drug use and hepatitis C or B virus co-infection were included in the model as fixed covariates. NLR, PLR and CD4 cell count were included as time-dependent covariates.

To evaluate whether the associations of NLR and PLR with risk of cancer were not linear, we also fitted time dependent Cox models with a cubic-spline for NLR and PLR, respectively. We used the Akaike’s information criterion to assess fitting of the models with linear and non-linear terms and to choose the number of spline knots.

Finally, we conducted sensitivity analyses. Particularly, we assessed the relations between NRL and PLR and cancer incidence i) using competing risk regression models with death from all causes as a competing event, ii) applying inverse probability weighted methods in order to adjust for selection bias due to losses to follow-up, iii) applying inverse probability weighted methods in order to adjust for selection bias due to missing values, iv) limiting the analysis to subjects enrolled from 1st January 2000, v) excluding the first year of follow-up; subjects who had diagnosis of NADC within 1 year were not included in such analysis, while the follow-up started 1 year later for everyone else, vi) using 1:1 nested case—control design matched on age, gender, date of enrolment and late presentation. The proportional hazards assumption was investigated for each covariate and globally by analyzing Schoenfeld residuals. We first produced the graphical plots and then carried out formal statistical tests of their independence over the rank transformation of time, but no departures from this assumption were found.

For statistical tests, P values lower than 0.05 were considered significant in two-tailed tests. All the computations were carried out using the Stata program for personal computer, version 12.0 (StataCorp, College Station, TX, USA).

## Results

A total of 13,559 HIV-positive subjects were included (73.3 % males, mean age ± standard deviation (SD) =36.0 ± 10.0) contributing 88,700.2 person-years (median 6.3 years of follow-up).

The patients’ characteristics at baseline are shown in Table 1. Hepatitis B or/and C virus co-infection were present in 43.4 %; 35.4 % of patients had intravenous drug use as risk factor for HIV infection acquisition, 33.4 % of patients was assuming ART and 25.8 % had undetectable HIV RNA (≤37 cp/ml). The median values (interquartile range) of NLR and PLR were 1.5 (1–2.2) and 109.9 (79.6–155.3), respectively.

**Table 1:** Patients’ characteristics at baseline

Characteristics	*n* (%)
	(*n* = 13,559)
Age, in years, median (IQR)	34.6 (28.4–41.8)
Male	9937 (73.3)
Immigrant	1667 (12.3)
Intravenous drug use	4799 (35.4)
HBV/HCV co-infection	5890 (43.4)
HIV-RNA viral load undetectable	3082 (25.8)
ART	4532 (33.4)
CD4 cell count, median (IQR)	378 (214–571)
0–49	611 (5.1)
50–99	633 (5.3)
100–199	1507 (12.56)
200–349	2747 (22.9)
350–499	2577 (21.5)
≥500	3932 (32.8)
CD4/CD8, median (IQR)	0.37 (0.20–0.61)
<0.3	2215 (39.7)
0.3–0.45	1119 (20.1)
≥0.45	2244 (40.2)
Lymphocytes, median (IQR)	1856 (1361–2390)
Neutrophils, median (IQR)	2694 (2000–3619)
Platelets, median (IQR)	210,999 (166,000–260,000)
NLR, median (IQR)	1.47 (1.03–2.17)
PLR, median (IQR)	109.9 (79.6–155.3)

Missing values: Immigrant 81 (0.6 %); HIV-RNA viral load undetectable 1609 (11.9 %), CD4 cell count 1552 (11.5 %), CD4/CD8 7981 (58.9 %), Lymphocytes 2607 (19.2 %); Neutrophils 4023 (29.7 %); Platelets 2553 (18.8 %); NLR 4978 (36.7 %); PLR 3107 (22.9 %)

*IQR* interquartile range, *HBV* hepatitis B virus, *HCV* hepatitis C virus, *ART* antiretroviral therapy, *NLR* neutrophil to lymphocytes ratio, *PLR* platelets to lymphocytes ratio

The cumulative probability of loss to follow-up at 3 years was 15.3 % (95 % CI, 14.6–15.9 %). Patients lost to follow-up, compared to those non-lost to follow-up, had a higher proportion of immigrants (21.1 % vs 10.9 %), intravenous drugs users (39.6 % vs 34.7 %), a lower proportion of HBV or HCV co-infection (35.4 % vs 44.8 %) and of subjects undergoing ART (26.1 % vs 34.6 %), and lower lymphocytes and platelets medians (Additional file 1: Table S1).

During the follow-up, 337 subjects had a first diagnosis of solid NADC, mainly in the liver (*n* = 75, 22.3 %), lung (*n* = 33, 9.8 %), rectum and anus (*n* = 27, 8.0 %) breast (*n* = 25, 7.4 %) and cutaneous melanoma (*n* = 17, 5.0 %) (Additional file 1: Table S2). The crude age- and gender-standardized incidence rates were 3.57 and 3.91 per 1000 person-years, respectively.

The predictive role of PLR and NLR for NADC incidence using time independent and time dependent Cox regression models is shown in Table 2. At univariate analysis, NLR showed no association with cancer incidence using time independent models, whereas it was associated with cancer risk when it was included as a continuous or dichotomized (higher than as compared to less than 1.47) variable in time dependent models. However, the association was not confirmed when fitting a multivariate time dependent model including also age, gender, CD4 cell count, presence of Hepatitis B or/and C virus co-infection and intravenous drug use. No association was found between PLR and cancer incidence using both time independent and time dependent models, when also considering PLR as continuous. Using a multivariate model, older age, presence of Hepatitis B or/and C virus co-infection and lower CD4 cell count were associated with solid NADC incidence (Additional file 1: Table S3).

**Table 2:** Associations of neutrophils to lymphocytes ratio and platelets to lymphocytes ratio with incidence of non-AIDS defining cancer using Cox proportional regression models

	Time independent model				Time dependent model			
	Univariate		Multivariate		Univariate		Multivariate	
	HR	95 % CI	HR	95 % CI	HR	95 % CI	HR	95 % CI
NLR								
1.47≤ vs >1.47	0.93	0.72–1.21	0.93	0.71–1.21	1.29	1.02–1.63	1.06	0.84–1.35
Continuous	1.03	0.93–1.14	1.03	0.93–1.14	1.14	1.08–1.21	1.06	0.99–1.15
PLR								
109.9≤ vs >109.9	0.83	0.66–1.05	0.81	0.64–1.04	0.98	0.77–1.22	0.78	0.61–1.01
Continuous	0.999	0.998–1.001	0.999	0.997–1.001	1.000	0.999–1.002	0.999	0.997–1.001

*HR* hazard ratio, *95 % CI* 95 % confidence interval, *NLR* neutrophils to lymphocytes ratio, *PLR* platelets to lymphocytes ratio

**Fig. 1:**
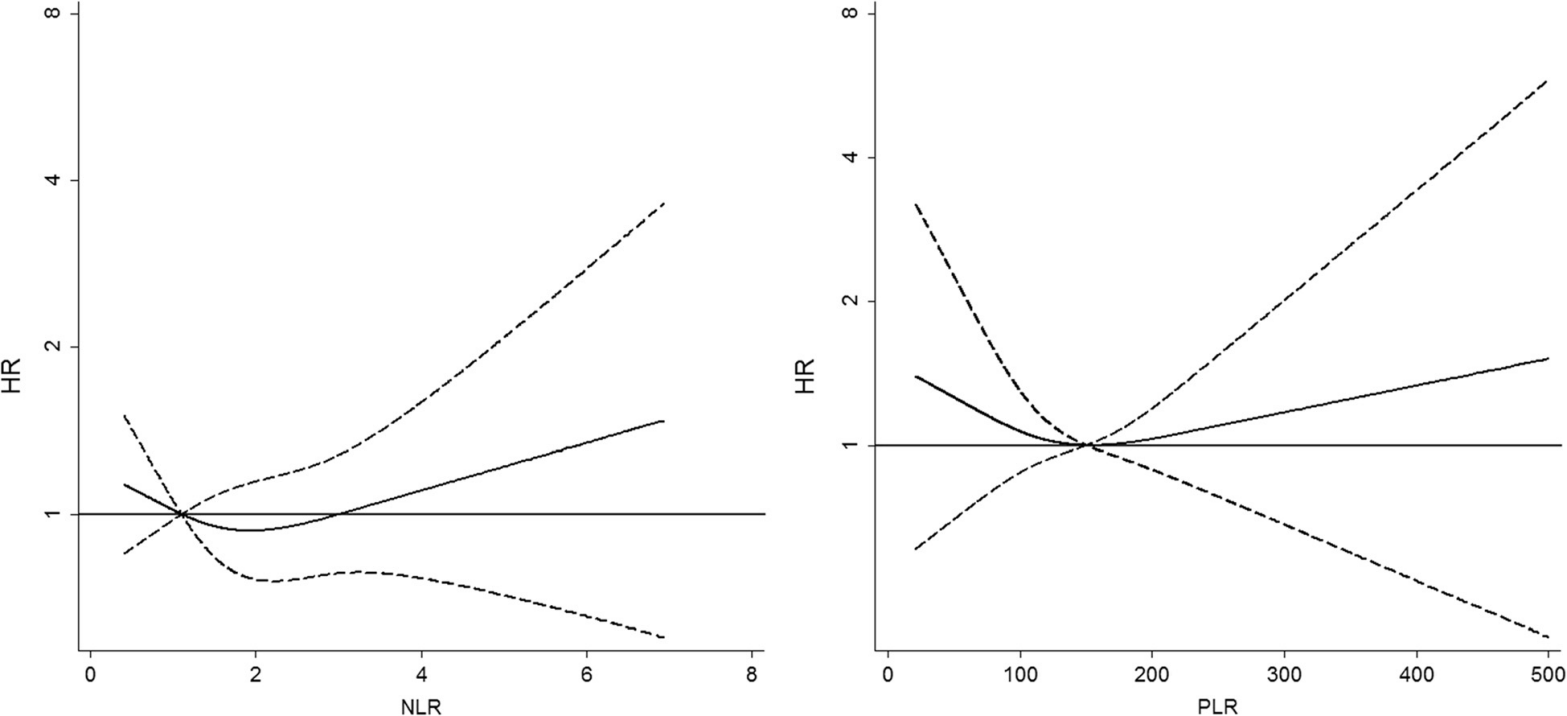
Hazard ratios (HRs) of non-AIDS defining cancer using cubic spline terms (solid lines) for neutrophils to lymphocytes ratio (NLR) and platelets to lymphocytes ratio (PLR) with three knots in Cox regression model. The reference values are 1.5 and 110, respectively. The 95 % confidence limits are shown as dashed lines. Vertical axes are on a logarithmic scale. Abbreviations: HR, hazard ratio; NLR, neutrophils to lymphocytes ratio; PLR, platelets to lymphocytes ratio

NLR and PLR were also evaluated in multivariate time-independent Cox regression models with restricted cubic-splines for these variables, which showed a modest, though not statistically significant increase of the hazard ratio for cancer with increasing serum values of the biomarkers (Fig. 1).

The results of the following sensitivity analyses were consistent with previous findings: (i) applying competing risk models, (ii) weighting models for losses to follow-up and (iii) missing values, (iv) limiting the analysis to patients enrolled after 2000 and naïve to antiretroviral therapy (6918 patients enrolled, with 123 NADC cases first diagnosed in follow-up), (v) excluding the first year of follow-up and (vi) using nested case—control design.

## Discussion

We did not observe an association between NLR and PLR serum levels and risk of solid NADC in HIV-infected patients after adjusting for age, gender, CD4 cell count, presence of HBV and or HCV co-infection and intravenous drug use. Both NLR and PLR values measured at baseline and during follow-up were not predictive of cancer development.

Chronic inflammation is believed to promote carcinogenesis and therefore to increase the risk of developing cancer. In last decade, several cohort studies among healthy people evaluated various inflammation markers, mainly interleukin-6 (IL-6), C-reactive protein (CRP), and tumor necrosis factor-α (TNF-α), as predictive of risk of overall cancer and particularly colorectal, breast, lung, liver and prostate cancer, with inconsistent findings [2–4, 20–26]. The different role of these biomarkers in the inflammation process, the single measurement of them in time, the relatively short follow-up of some studies are possible explanations for these discrepancies. Furthermore, some authors found that only a small part of the total inflammation biomarkers tested was associated with risk of cancer and proposed a combination of some of them in a predictive score [26]. Therefore, the negative findings of our study on the possible predictive role of NLR and PLR in HIV positive subjects, who may suffer from chronic inflammation due to their disease and treatment, when also considering repeated measures of these biomarkers during follow-up, are not really surprising compared to the mentioned studies. It is of note, however, that no study investigated the role of NLR and PLR as predictors of cancer risk in the general population so far, to our knowledge.

In our study we could not evaluate the association of NLR and PLR with incidence of specific cancer types or sites because of the relatively small number of patients who developed cancer in the follow-up, mainly due to the low mean age of HIV patients at enrollment and relatively short duration of the follow-up in the MASTER cohort. Therefore, our findings do not exclude the possibility that the levels of these inflammatory markers may be predictive of the risk of HIV positive subjects to develop specific neoplasms.

The main strengths of this study are: (i) the fair size of the cohort for evaluating risk of overall cancer, (ii) the completeness of data on cancer occurrence, (iii) the lack of selection in patients included in the cohort, which provides an unbiased “real world” scenario of the clinical practice and epidemiological pattern of HIV infection and AIDS disease throughout Italy, (iv) the availability of repeated measures of NLR and PLR in time, and (v) the analysis of time dependent and time independent regression models for evaluating these biomarkers at baseline and during the follow-up. This study has some limits, too, mainly the small number of cancer cases, the lack of data on the main risk factors for cancer and on the clinical stage or other parameters of severity of cancer at diagnosis.

Around one third of the subjects (36 %) were lost to follow-up at 3 years. They differed from those unlost for various characteristics. However, there were no significant differences either in the variables predictive of cancer risk by multivariate model apart from HBV/HCV coinfection, or in NLR or PLR values. Therefore, it is unlikely that loss to follow-up may have caused bias in the main findings of our study.

## Conclusions

This study does not sustain the hypothesis that NRL and PLR, two simple and low-cost inflammatory parameters associated with risk of death in people with cancer, may be also useful to predict cancer occurrence in HIV positive subjects.

## Additional file

Additional file 1: Table S1. Patients’ characteristics according to lost to follow-up at 3-years. **Table S2.** Distribution of non-AIDS defining cancer. **Table S3.** Multivariate analysis: time dependent Cox regression model. Variables included in the full model. (DOCX 20 kb)

## Abbreviations

NLR: Neutrophils to lymphocytes ratio
PLR: Platelet to lymphocytes ratio
NADC: Non-AIDS defining cancer
ADC: AIDS defining cancer
ART: Antiretroviral therapy
ICD: International classification of diseases
HR: Hazards ratio
SD: Standard deviation
