# Supplementary material for: The predictive role of NLR and PLR for solid non-AIDS defining cancer incidence in HIV-infected subjects: a MASTER cohort study
Source: Infect Agent Cancer. 2015 Oct 5;10:34. doi: 10.1186/s13027-015-0032-y (PMC4593208; doi:10.1186/s13027-015-0032-y)
Supplement: Additional file 1: Table S1. — Patients’ characteristics according to lost to follow-up at 3-years. Table S2. Distribution of non-AIDS defining cancer. Table S3. Multivariate analysis: time dependent Cox regression model. Variables included in the full model. (DOCX 20 kb) [file 13027_2015_32_MOESM1_ESM.docx]

**Additional file 1**

**Table S1**. Patients’ characteristics according to lost to follow-up at 3-years.

| **Characteristics** | **Not lost** | **Lost** | **p-value** |
| --- | --- | --- | --- |
|  | **n (%)** | **n (%)** |  |
|  |  |  |  |
| Age, in years, median (IQR) | 34.5 (28.3-41.9) | 35.1 (29.1-41.4) | NS |
| Male | 8561 (73.5) | 1376 (72.3) | NS |
| Immigrant | 1269 (10.9) | 395 (21.1) | <0.001 |
| Intravenous drug use | 4046 (34.7) | 753 (39.6) | <0.001 |
| HBV/HCV co-infection | 5216 (44.8) | 674 (35.4) | <0.001 |
| HIV-RNA viral load undetectable | 2672 (25.9) | 410 (25.4) | NS |
| ART | 4036 (34.6) | 496 (26.1) | <0.001 |
| CD4 cell count, median (IQR) | 378 (216-573) | 375 (206-559) | NS |
| 0-49 | 507 (4.9) | 504 (31.3) |  |
| 50-99 | 553 (5.3) | 355 (22.1) |  |
| 100-199 | 1299 (12.5) | 358 (22.3) |  |
| 200-349 | 2389 (23.0) | 208 (12.9) |  |
| 350-499 | 2222 (21.4) | 80 (4.5) |  |
| ≥ 500 | 3428 (33.0) | 104 (6.5) |  |
| CD4/CD8, median (IQR) | 0.37 (0.20-0.61) | 0.41 (0.22-0.65) | 0.016 |
| <0.3 | 1902 (40.5) | 313 (35.6) |  |
| 0.3-0.45 | 939 (20.0) | 180 (20.5) |  |
| ≥0.45 | 1857 (39.5) | 387 (44.0) |  |
| No. Lymphocytes, median (IQR) | 1861 (1370-2400) | 1822 (1327-2330) | 0.037 |
| No. Neutrophils, median (IQR) | 2699 (2000-3610) | 2650 (1970-3660) | NS |
| No. Platelets, median (IQR) | 211000 (167000-260000) | 207999 (158999-255999) | 0.005 |
| NLR, median (IQR) | 1.47 (1.02-2.15) | 1.53 (1.05-2.24) | NS |
| PLR, median (IQR) | 109.9 (79.6-155.2) | 110.0 (79.6-155.7) | NS |

**Abbreviations:** IQR, interquartile range; NS, not statistically significant (p<0.05); HBV, hepatitis B virus; HCV, hepatitis C virus; ART, antiretroviral therapy; NLR, neutrophil to lymphocytes ratio; PLR, platelets to lymphocytes ratio.

**Table S2**. Distribution of non-AIDS defining cancer.

| **Cancer Type or site (ICD-10)** | **n (%)**  **(n = 337)** |
| --- | --- |
| Tongue and lingual tonsil (C01-C02) | 4 (1.2) |
| Stomach (C16) | 11 (3.3) |
| Colon (C18) | 10 (3.0) |
| Rectum-Anus (C19-21) | 75 (22.3) |
| Liver, primary (C22) | 27 (8.0) |
| Larynx (C32) | 5 (1.5) |
| Lung, bronchus and trachea (C33-34) | 33 (9.8) |
| Melanoma (C43) | 17 (5.0) |
| Breast (C50) | 25 (7.4) |
| Vagina and vulva (C51-52, 57) | 5 (1.5) |
| Penis (C60) | 5 (1.5) |
| Prostate (C61) | 6 (1.8) |
| Testis (C62) | 12 (3.6) |
| Kidney (C64-66, C68) | 7 (2.1) |
| Bladder (C67) | 9 (2. 7) |
| Brain and CNS (C70-72) | 3 (0.9) |
| Thyroid (C73) | 7 (2.1) |
| Others | 76 (22.6) |

**Abbreviation**: ICD: international classification of diseases

**Table S3**. Multivariate analysis: time dependent Cox regression model. Variables included in the full model.

| **Variables** | **HR** | **95% CI** | **p-value** |
| --- | --- | --- | --- |
| Male vs Female | 0.90 | 0.69-1.19 | NS |
| Age | 1.06 | 1.05-1.08 | <0.001 |
| HBV/HCV co-infection | 1.49 | 1.08-2.06 | 0.016 |
| CD4 cell count | 0.998 | 0.998-0.999 | <0.001 |

**Abbreviations:** HR, hazard ratio; 95% CI, 95% confidence interval; NS, not statistically significant (p<0.05).
